# *Brugia malayi *Asparaginyl - tRNA Synthetase Stimulates Endothelial Cell Proliferation, Vasodilation and Angiogenesis

**DOI:** 10.1371/journal.pone.0146132

**Published:** 2016-01-11

**Authors:** Jeeva Jothi D, Muthu Dhanraj, Shanmugam Solaiappan, Sanjana Sivanesan, Michael Kron, Anuradha Dhanasekaran

**Affiliations:** 1 Center for Biotechnology, Anna University, Chennai-25, Tamil Nadu, India; 2 Sri Ramachandra Medical College, Porur, Chennai, India; 3 Biotechnology and Bioengineering Center, the Department of Medicine, Division of Infectious Diseases, Medical College of Wisconsin, Milwaukee, Wisconsin, United States of America; University of Illinois at Chicago, UNITED STATES

## Abstract

A hallmark of chronic infection with lymphatic filarial parasites is the development of lymphatic disease which often results in permanent vasodilation and lymphedema, but all of the mechanisms by which filarial parasites induce pathology are not known. Prior work showed that the asparaginyl-tRNA synthetase (BmAsnRS) of *Brugia malayi*, an etiological agent of lymphatic filariasis, acts as a physiocrine that binds specifically to interleukin-8 (IL-8) chemokine receptors. Endothelial cells are one of the many cell types that express IL-8 receptors. IL-8 also has been reported previously to induce angiogenesis and vasodilation, however, the effect of BmAsnRS on endothelial cells has not been reported. Therefore, we tested the hypothesis that BmAsnRS might produce physiological changes in endothelial by studying the *in vitro* effects of BmAsnRS using a human umbilical vein cell line EA.hy926 and six different endothelial cell assays. Our results demonstrated that BmAsnRS produces consistent and statistically significant effects on endothelial cells that are identical to the effects of VEGF, vascular endothelial growth factor. This study supports the idea that new drugs or immunotherapies that counteract the adverse effects of parasite-derived physiocrines may prevent or ameliorate the vascular pathology observed in patients with lymphatic filariasis.

## Introduction

Lymphatic filariasis is a high morbidity infectious disease that affects more than 200 million people worldwide. Filariasis is caused by filarial nematode parasites, mainly *Wuchereria bancrofti* and *Brugia* species, which cause chronic infection and frequently, the clinical manifestation known as elephantiasis. The pathogenesis of lymphatic filariasis results from a complex interplay between the parasite and the immune response of the host, as well as superimposed microbial infections [1,2].

Aminoacyl-tRNA synthetases (AARSs) are a family of evolutionarily ancient enzymes responsible for both primary and secondary biological activities in all prokaryotes and eukaryotes [3]. AARSs catalyze the aminoacylation or “charging” of isoacceptor tRNAs with the correct amino acid so that protein synthesis can occur. In 1998 Weiner suggested that as primordial AARS evolved over time, catalytic domains have “broken loose” to perform unexpected catalytic and regulatory functions [4]. Numerous secondary biological activities for specific eukaryotic AARSs have been reported [5,6]. These unexpected secondary activities include transcriptional regulation, mitochondrial RNA splicing, control of cell growth, and cytokine or chemokine-like activity. Six human AARS function as autoantigens in a rare subset of human autoimmune diseases known as “anti-synthetase syndromes” [7–9]. The six autoantigenic human AARSs display unique protein domains that interact with specific chemokine receptors. “Physiocrine” is a new term that is used to describe select eukaryotic AARS that demonstrate novel cell signaling roles or immunologically important secondary activities and thus may contribute to immunopathology.

In *Brugia malayi and Wuchereria bancrofti*, the asparaginyl-tRNA synthetase (AsnRS) proteins are identical at the amino acid level and exist as multi-copy genes encoding an immunodominant antigen that produces a strong antibody response in the serum of humans with lymphatic filariasis [10,11]. More recent studies demonstrated that the filarial AsnRS is ten times more highly expressed than other AARSs in the parasite, and is actively excreted and secreted from adult worms [12,13]. The atomic structure of the complete *B*. *malayi* AsnRS has been solved and consists of two structured domains: (1) a novel 110 amino acid amino terminal domain in which 80 amino acids fold the same way that interleukin-8 (IL-8) folds to interact with extracellular loops of the G-protein coupled IL-8 receptors, and (2) a 438 amino acid catalytic domain [14,15]. *In vitro* rBmAsnRS promoted what were first thought to be pro-inflammatory activities, such as chemotaxis of cells that express IL-8 receptors [16]. *In vivo* however, intraperitoneal administration of rBmAsnRS yields potent anti-inflammatory properties that resolves gut pathology in the T-cell transfer mouse model of colitis [17].

Endothelial cells are one of the many cell types that express IL-8 receptors and thus in theory may be influenced by the filarial AsnRS [18,19]. IL-8 has been reported previously to induce angiogenesis by stimulating the production of VEGF, vascular endothelial growth factor, and involves the NFkB signal transduction pathway. Although it is known that BmAsnRS actives IL-8 receptors and the NFkB pathway, the effect of rBmAsnRS has never been studied in endothelial cells. Therefore, we postulated that physiological concentrations of BmAsnRS might alter endothelial cell function, either stimulating or inhibiting cellular activity. We tested this hypothesis by comparing the effects of VEGF to those of BmAsnRS in six different models of endothelial cell function.

## Materials and Methods

### Chemicals

Isopropyl-β-D-thiogalactopyranoside, L-Asparagine, Sodium ATP, Pyrophosphatase, malachite green solution and 4,6-diamidino-2-phenylindole dihydrochloride (DAPI) were purchased from Sigma Chemical Co. Dulbecco’s Modified Eagle Medium (DMEM), fetal bovine serum (FBS), penicillin, and streptomycin were purchased from PAN Biotech Aiden Bach, Bavaria. Matrigel was purchased from BD Biosciences. MTT reagent and all other chemicals were of the reagent grade and were obtained commercially.

### Endothelial cell culture

EA.hy926 endothelial cells were a kind gift from Dr. C.J.S. Edgell, Tissue Culture Facility, UNC Lineburger Comprehensive Cancer Center, University of North Carolina, Chapel Hill. Use of EA.hy926 cell line was approved by Institutional Biosafety and Ethical Committee of AU-KBC Research Centre, Chennai on 13th October 2012. EA.hy926 cells are an immortalized human umbilical vein-derived cell line that retains characteristics of vascular endothelial cells [20]. Cells were cultured in DMEM supplemented with 10% glucose, 10% FBS (v/v), 1% penicillin (w/v) and streptomycin (w/v).

### Expression and purification of recombinant, biologically active BmAsnRS

The full length 548 amino acid wild type BmAsnRS was expressed using the pET15B *E*. *coli* expression system as previously described [12–17]. Luria Bertani (LB) broth was used for the growth of *E*. *coli* strains. A pET15B construct containing the full length BmAsnRS cDNA was transformed into *E*. *coli* BL21 (DE3) cells and BmAsnRS expression was induced with IPTG. Soluble recombinant BmAsnRS (rBmAsnRS) of the expected molecular mass was produced and purified by IMAC (immobilized metal-affinity chromatography) by virtue of N-terminal hexa-histidine tag. The rBmAsnRS protein was eluted with 150 mM imidazole in elution buffer at pH 7.0. The fraction containing protein were pooled and dialyzed overnight at 4°C in 20mM HEPES pH 7. The production of intact rBmAsnRS protein was visualized on 12% sodium dodecyl sulfate polyacrylamide gels. Correct folding, dimerization and enzymatic activity of the rBmAsnRS was confirmed before use in endothelial cell assays using a published pre-transfer editing assay [21]. Briefly, when property folded and dimerized, rBmAsnRS catalyzes the production of pyrophosphate in the first step of the two step aminoacylation reaction. Bacterial pyrophosphatase is included in the reaction buffer and serves to convert pyrophosphate to phosphate (Pi). Pi is detectable quantitatively using Malachite Green reagent. rBmAsnRS is the same protein as the wild type BmAsnRS.

### Endothelial Cell Assays

#### Proliferation assay

The yellow dye (3-(4, 5-dimethylthiazol-2-yl)-2, 5-diphenyltetrazolium bromide) MTT, is reduced by metabolically proliferating cells resulting in a purple formazan that can be quantified spectrophotometrically [22]. EA.hy926 cells were grown to 70% confluence in 200μl media per well in a 96 well plate. Cells were incubated at 37°C, 5% CO_2_ overnight to allow the cells to adhere to the wells. 1–10 ng/mL of rBmAsnRS or VEGF was added to each well with cells to determine its effects on proliferation. 10 ng/mL VEGF was used as positive control. Cells were incubated at 37°C, 5% CO_2_ for 24 hours. Increased production of purple formazan was an indicator of cell proliferation and was measured with an ELISA plate reader at 570 nm.

#### Chemotaxis

Trypsinized EA.hy926 cells were used for migration assays in standard Boyden's chambers. EA.hy926 cells were loaded in the upper well of a Boyden chamber with DMEM and the lower well was filled with different concentrations of rBmAsnRS (1–10 ng/mL) in DMEM. 10 ng/ml VEGF was used as a positive control. The chambers were then incubated at 37°C, 5% CO_2_ for 2 hours. Cells that migrated across the membrane and adhered to the lower surface of the membrane constitute chemotaxis. After incubation, the polycarbonate membrane was fixed and stained with DAPI, a fluorescent nuclear probe. Endothelial cell chemotaxis was quantified by counting the number of migrated cells adherent to the lower surface of the membrane. The migrated cells were counted at 20 times magnification using a fluorescent microscope (Olympus IX71).

#### Endothelial cell ring formation

In this assay stimulated endothelial cells can form ring-like structure within 2 hours. EA.hy926 cells were seeded onto collagen-coated 12-wellplates (1 X 10^5^ cells per well). After 4 hours of incubation at 37°C, 5% CO_2_, the media was changed to media supplemented with different concentrations of rBmAsnRS (1–10 ng/mL). VEGF (10 ng/mL) was used as a positive control. The cells were incubated with rBmAsnRS or VEGF for two hours at 37°C, 5% CO_2_. The number of rings formed under the influence of VEGF and rBmAsnRS were counted at 20 X magnifications using a bright field phase contrast microscope [23].

#### Three dimensional tube formation

Rapidly growing endothelial cells can be induced to form three dimensional tube-like structures *in vitro*. 10^4^ EA.hy926 cells were grown in 24-well plates coated with Matrigel™. Cells were incubated with rBmAsnRS (5 and 10 ng/mL) and VEGF (10 ng/mL) was used as a positive control. The number of endothelial cell tubes formed were counted after 24 hours [24].

#### Angiogenesis in chicken chorioallantoic membrane

Embryonated chicken eggs were collected from the Centre for Animal Health Studies, TANUVAS, Madhavaram Milk Colony, Chennai. The eggs were broken and gently placed in Petri dishes under sterile conditions. Paper discs were soaked in different concentrations of rBmAsnRS (2.5–10 ng/mL) or VEGF (10 ng/mL) and one disc were placed on each egg yolk and incubated for another twenty-four hours. Serial images of vessels were taken in 2X and 4X magnification using Nikon Cool Pix camera (Olympus India Pvt Ltd, New Delhi, India) adapted to a stereomicroscope at 0^th^, 12^th^ and 24^th^ hours of incubation. The length of specific vessels was documented using Scion Image, Release Alpha 4.0 3.2 and Adobe Photoshop version 6.0 and analyzed using Angioquant software [25,26].

#### Vasodilation in chicken chorioallantoic membranes

Embryonated chicken eggs were collected from the Centre for Animal Health Studies, TANUVAS, Madhavaram Milk Colony, Chennai. Briefly, the egg shell of fifth day eggs was gently cut open to expose the area vasculosa for video recording. Paper discs soaked in various rBmAsnRS concentrations (2.5–10 ng/mL) or VEGF (10 ng/mL) and individual discs were placed directly on the blood vessels and recorded for 20 minutes time interval. 0, 5, 10, 15 and 20 minutes images were used to calculate the dimensions of a fixed portion of blood vessel. The change in width of vessels was monitored for each concentration of rBmAsnRS and VEGF. The widths of blood vessels were measured during 20 minute videotaped experiments using Image J analysis software (Release 4.0 3.2).

### Statistical analysis

All statistical analyses used GraphPad Prism software version 5. Numerical data were presented as means ± SEM from three to five different replicates in all the experiments. The difference between groups was analyzed using ANOVA followed by TUKEYs test when permitted. Values for P< 0.05 were considered as statistically significant.

## Results

### Purification of rBmAsnRS

**Fig 1:**
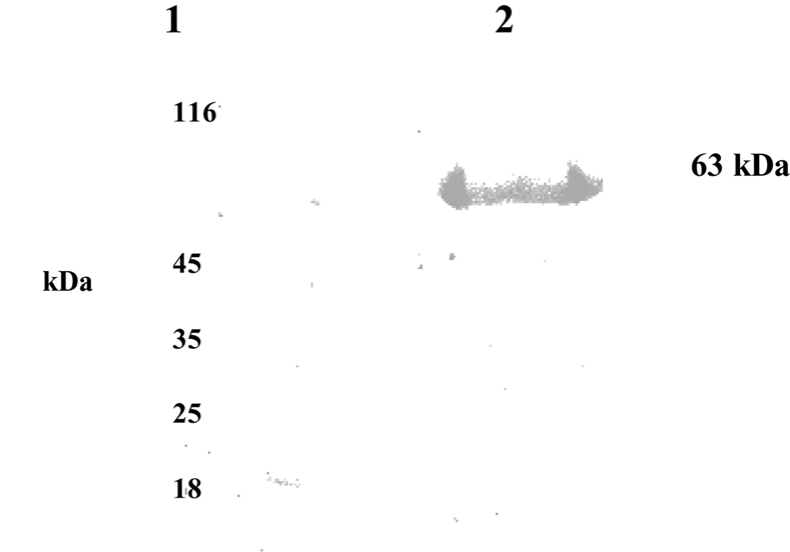
Hexa-histidine tagged rBmAsnRS was purified from the sonicated supernatant of IPTG induced *E*. *coli* cultures expressing the full length wild type BmAsnRS cDNA. Purified rBmAsnRS was subjected to electrophoresis in a 12%SDS-polyacrylamide gel and visualized by staining with Coomassie blue R250. Lane 1: molecular weight markers. Lane 2: purified rBmAsnRS.

The distribution of induced rBmAsnRS was studied in the supernatant vs cellular pellet after sonication of cultures in the equilibration buffer (pH 8.0). rBmAsnRS was present at higher concentration in the soluble fraction which facilitated its purification by IMAC by virtue of N-terminal hexa-histidine tag (**Fig 1**). rBmAsnRS protein was eluted from histidine tagged substrate with 150 mM imidazole elution buffer at pH 7.0. The fractions containing protein were pooled and dialyzed overnight at 4°C in 20mM HEPES pH 7.5 to remove imidazole. Only after purified rBmAsnRS was documented to have normal enzymatic activity in the pre-transfer editing assay, was the rBmAsnRS used in subsequent endothelial cell assays [21].

### rBmAsnRS and VEGF induce endothelial cell proliferation

**Fig 2:**
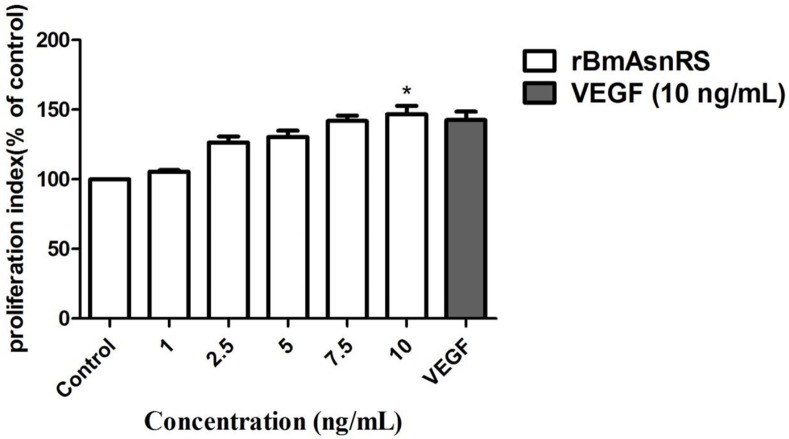
The effect of rBmAsnRS and VEGF on cell viability was determined using the MTT assay. EA.hy962 cells were incubated with various concentrations of rBmAsnRS for 24 hours and compared to the effects of 10 ng/ml VEGF. Data are presented as mean ± SEM from five replicates. Proliferation index (% of control) of 1 ng/ml was 0.8996, 10 ng/ml was 1.33263 and 10 ng/ml VEFG was 1.30298. Asterisk (*) denotes significant differences of p<0.0001 for control vs 10 ng/ml rBmAsnRS and 10 ng/ml VEGF.

The MTT assay was used to measure EA.hy926 endothelial cell proliferation in response to various concentrations of enzymatically active rBmAsnRS (1–10 ng/mL) or VEGF (10ng/mL). rBmAsnRS and VEGF induced significant and equally strong cell proliferation (*P≤*0.0001) at 10 ng/ml (**Fig 2**).

### rBmAsnRS and VEGF promote chemotaxis of EA.hy926 cells

**Fig 3:**
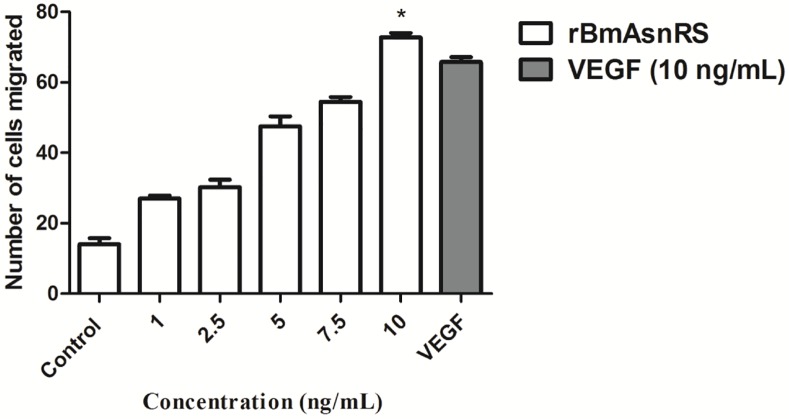
The effect of rBmAsnRS and VEGF on EA.hy926 cell migration was determined in Boyden chamber experiments. Data are presented as means ± SEM. The number of cells migrated in response to 1 ng/ml rBmAsnRS was 14, and 10 ng/ml rBmAsnRS caused the greatest migration (72.75) similar to 10 ng/ml VEGF. Asterisk (*) denotes significant differences (p<0.0001) for negative control vs 10 ng/ml rBmAsnRS and VEGF.

The chemotactic migration of endothelial cells is a key event in angiogenesis and thus we measured this phenomenon using Boyden's chambers. 1–10 ng/mL concentrations of rBmAsnRS were used to evaluate the effect of rBmAsnRS on EA.hy926 cells. Analysis of Boyden's chamber experiment showed that rBmAsnRS induced a concentration dependent migration of cells which at 10 ng/ml was equal to migration induced by 10ng/ml VEGF (**Fig 3**).

### rBmAsnRS and VEGF induce ring formation by EA.hy926 cells

**Fig 4:**
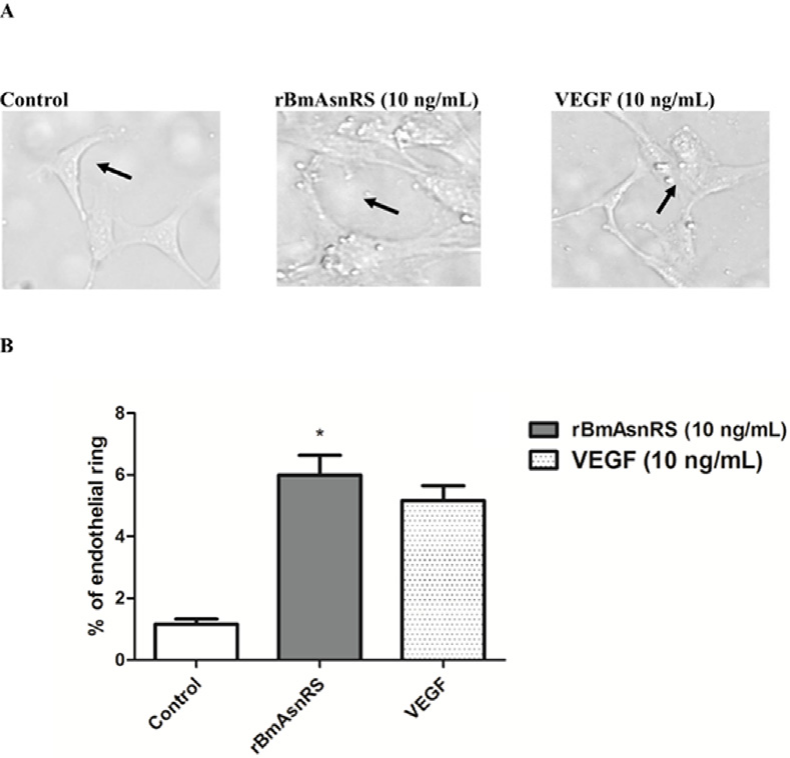
The effect of rBmAsnRS and VEGF in endothelial ring formation. Fig 4A. Photomicrographs show the morphology of endothelial rings (arrows) formed by EA.hy926 cells in response to stimulation with 10 ng/ml rBmAsnRS and 10 ng/ml VEGF. Fig 4B. Graphical representation of the induction of endothelial rings in response to rBmAsnRS and VEGF. Data are means ± SEM. The percentage of cells forming endothelial rings in 10 ng/ml rBmAsnRS and 10 ng/ml VEGF were similar and both were significantly greater than the PBS control. Asterisk (*) denotes p<0.0001.

In this assay endothelial cells form ring-like structure within 2 hours under the influence of VEGF (positive control). The number of rings formed under the influence of 10 ng/ml VEGF and 10 ng/ml rBmAsnRS were counted at 20 X magnifications using a bright field phase contrast microscope. The percent of endothelial cell rings formed was the same in VEGF and rBmAsnRS treated cells (**Fig 4A and 4B**). **Fig 4A** shows representative photomicrographs of rings. **Fig 4B** is a graphical representation of differences in the number of formed in response to rBmAsnRS, VEGF and control.

### rBmAsnRS and VEGF induce tube formation by EA.hy926 cells in Matrigel

**Fig 5:**
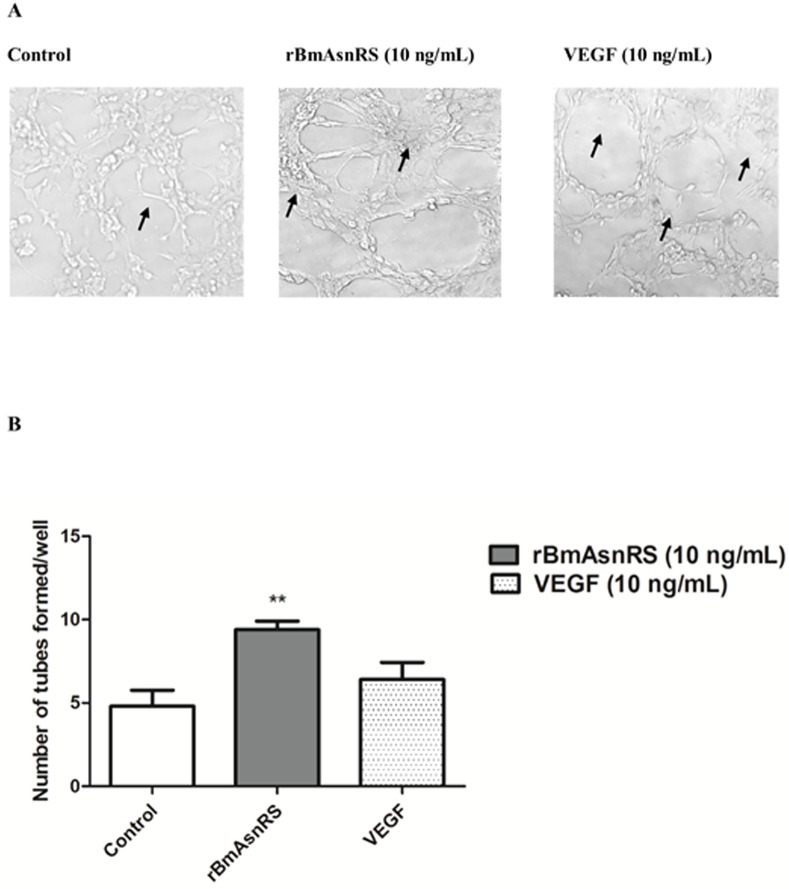
The effect of rBmAsnRS and VEGF on tube formation. Fig 5A. Images indicate with arrows the endothelial tube formation in negative control, 10 ng/ml rBmAsnRS and 10 ng/ml VEGF groups. Photomicrographs were taken with 20X magnification under an inverted bright field microscope. Fig 5B. Graphical representation of the number of tubes formed by EA.hy926 cells exposed to negative control, 10 ng/ml rBmAsnRS or 10 ng/ml VEGF for 24 hours in Matrigel-coated 24-well plates. Data are presented as means ± SEM. Asterisk (*) denotes statistically significant differences compared to negative control.

To examine the effects of rBmAsnRS on endothelial tube formation, we used a cell-based model of tube formation starting with a monolayer culture of immortalized EA.hy926 cells in solid Matrigel (**Fig 5A and 5B**). The number of endothelial tubes formed were measured at rBmAsnRS concentrations of 5ng/ml and 10 ng/ml for a period of 24 hours incubation. The number of tubes formed per well by 10 ng/ml rBmAsnRS was significantly greater than negative control and the same as the number of tubes induced by 10 ng/ml VEGF. **Fig 5A** shows photomicrographs of representative tubes formed. **Fig 5B** is a graphical representation of new tube formation.

### rBmAsnRS and VEGF induce lengthening of vessels in an *ex vivo* model

**Fig 6:**
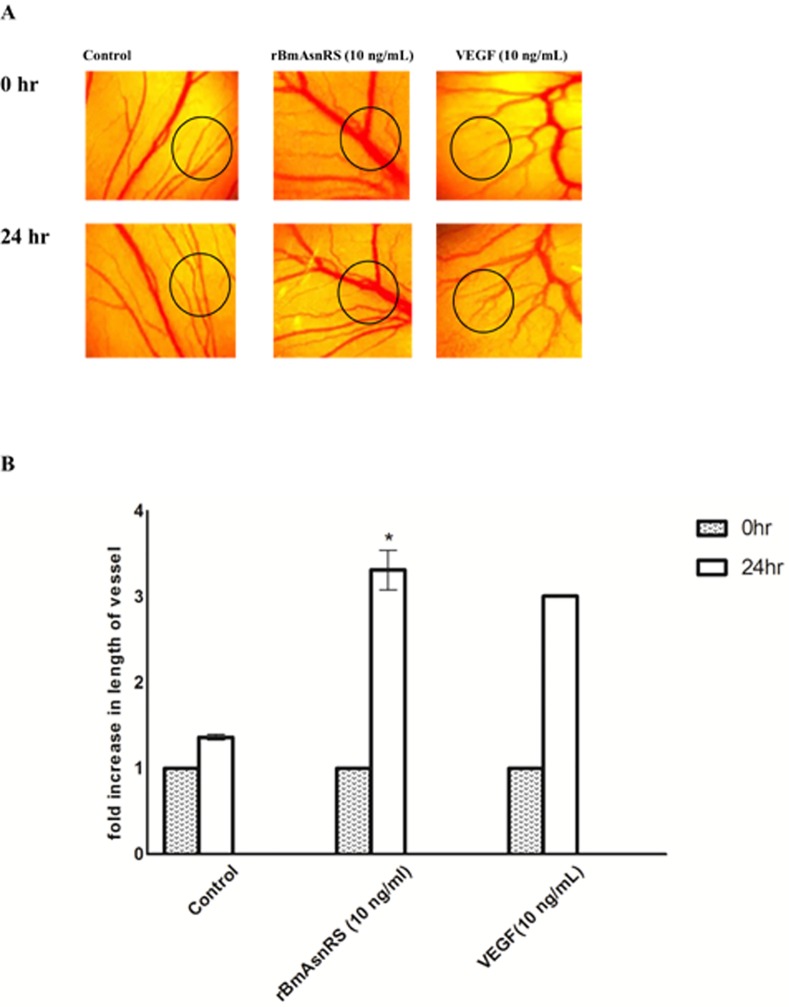
The effect of rBmAsnRS and VEGF *in ex vivo* angiogenesis. Fig 6A. Photomicrographs denote areas with angiogenesis induced by 10 ng/ml rBmAsnRS and 10 ng/ml VEGF. Angiogenesis was induced by placing sterile paper discs containing rBmAsnRS, VEGF or negative control (PBS) on the egg yolk vascular bed for 30 min, then incubated for another 24 hours. Images are representative of five different experiments. Fig 6B. Graphical representation of the fold increase in vessel length induced by 10 ng/ml rBmAsnRS and 10 ng/ml VEGF. Data are presented as means ± SEM of five experiments. The fold increase in length of blood vessel in 10 ng/ml rBmAsnRS and 10 ng/ml VEGF were significantly greater than negative controls. Asterisk (*) denotes statistically significant difference (p<0.005).

The effects of 10 ng/mL rBmAsnRS and 10 ng/mL VEGF on the whole vascular bed of the developing chick embryo were explored using a modified chicken chorioallantoic membrane assay. PBS was used as a negative control. A statistically significant increase in vascularity (vessel length) was induced by 10 ng/mL rBmAsnRS and 10 ng/mL VEGF after 24 hours. (**Fig 6A and 6B). Fig. A** shows representative photomicrographs of vessels. The statistically greater vessel length is more clearly quantified by measurement of the fold increase in vessel length as shown in **Fig 6B**.

### rBmAsnRS and VEGF induce vasodilation in chick embryo chorioallantoic membranes

**Fig 7:**
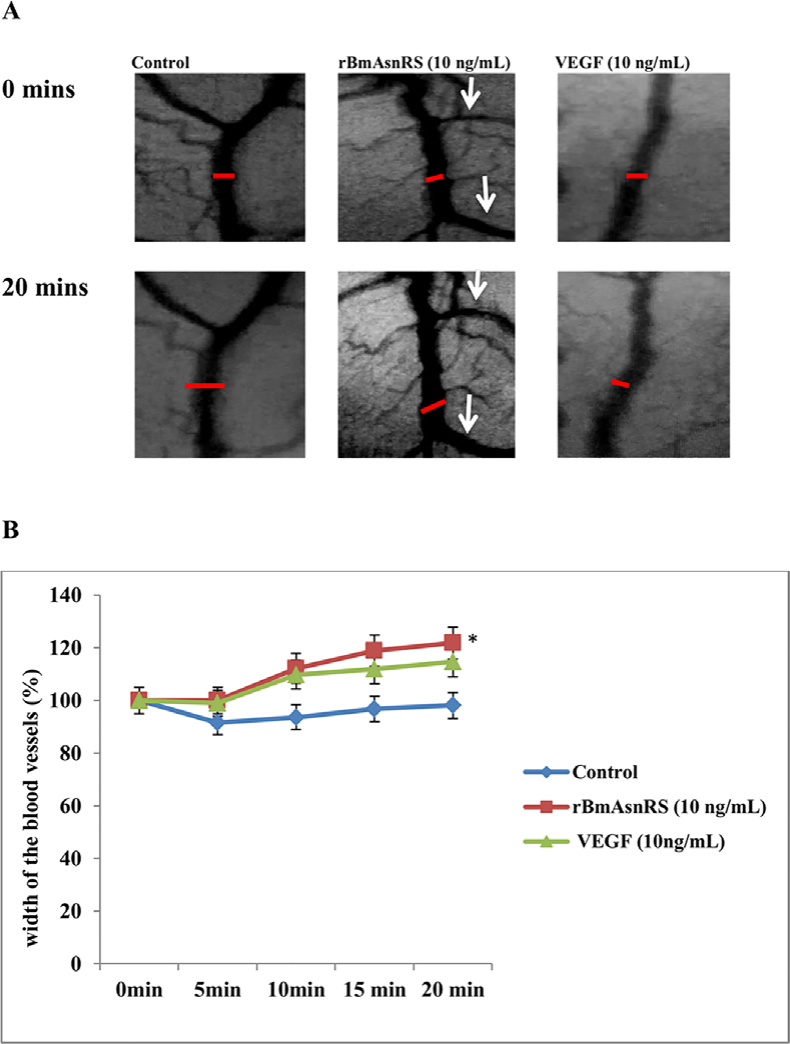
The effect of rBmAsnRS and VEGF on vasodilation of capillary blood vessels. Fig 7A. Images represent the change in capillary width induced by 10 ng/ml rBmAsnRS and 10 ng/ml VEGF. Images were captured after 20 minutes observation and are representative of 3 sets of experiments. Fig 7B. Graphical representation of changes in vessel width induced by rBmAsnRS and VEGF. Data are presented as means ± SEM. rBmAsnRS and VEGF treated blood vessels were both significantly dilated compared to controls. Asterisk (*) indicates statistically significant difference between control vs rBmAsnRS or VEGF (p < 0.005.).

The effect of 10 ng/ml rBmAsnRS and 10 ng/ml VEGF were compared in the chick chorioallantoic membrane assay. 1X PBS was used as the negative control. Whatman no.1 sterile discs were saturated with rBmAsnRS, VEGF or PBS and individual discs were then placed on the chick vascular beds in triplicate. Videos of capillary beds were taken from zero to twenty minutes of the same field to monitor changes in vessel diameters (**Fig 7A and 7B**). This experiment showed a subtle but consistent and statistically significant increase in vessel diameter was induced by both VEGF and rBmAsnRS. **Fig 7A** shows photomicrographs of representative vessel changes. **Fig 7B** is a graphical representation of vessels width changes that clearly demonstrates the statistically significant increase in vessel diameters induced by both rBmAsnRS and VEGF. The complete time lapse video documentation of blood vessel responses is available as Supplementary Material (S1–S3 Videos).

In summary, the stimulatory effect of rBmAsnRS on EA.hy926 cells and in chick chorioallantoic membrane assays was consistently observed and equal to the effects of VEGF. The concentrations of rBmAsnRS and VEGF used to demonstrate these effects were in the same range reported for typical chemokine-receptor mediated phenomenon observed in human leukocytes [16].

## Discussion

The present study adds several additional activities to the known repertoire of the filarial nematode physiocrine, rBmAsnRS–endothelial cell proliferation, migration, ring and tube formation, vasodilation and angiogenesis. In previous research, rBmAsnRS was shown to exhibit several different *in vitro* and *in vivo* activities–leukocyte chemotaxis, activation of MAP kinases and calcium transients via G protein coupled IL-8 receptors (CXCR1 and CXCR2); endogenous ATPase activity and biosynthesis of the diadenosine oligophophate Ap3A (diadenosine triphosphate) which also induces vasodilation in endothelial cells via a subset of purine receptors [13,27]. The present study also demonstrates that rBmAsnRS and VEGF are equally strong at stimulating proliferation and migration of EA.hy926 cells to form ring-like structures in cell monolayers. Both rBmAsnRS and VEGF also induce ring-like structures to form endothelial cell tubes when grown in solid (Matrigel) media. These results are consistent with the IL-8 mediated angiogenic effects on endothelial cells reported previously by other groups. But this study is novel in that it is the first report of a filaria-derived AARS that mimics IL-8 in angiogenesis. In addition, the stimulatory effects of rBmAsnRS in egg yolk models demonstrated it can promote angiogenesis in an *ex vivo* model and at the peripheral capillary ends of the vascular bed.

Angiogenesis is the multistep process of generating new blood vessels derived as extensions from the existing vasculature [23–26, 28–32]. The molecular process of endothelial cell migration/chemotaxis involves changes in cell adhesion, permeability, signal transduction, and reorganization of the cytoskeleton. Upon activation of endothelial cells by inflammatory mediators such as IL-8 and VEGF, the endothelium becomes a major participant in the generation of an inflammatory response. In 1980 Folkman and Haudenschild first observed angiogenesis *in vitro* and reported that endothelial cells could be induced to form ring-like structures in cell monolayers [24]. Those cells in turn were capable of proliferation, differentiation and migration into nearby connective tissues, and eventually were capable of forming a “sprout,” or cord of endothelial cells with lumens. Ultimately those sprouts become venules and joined to form a group of blood vessels. These emerging vessels then recruit peri-endothelial cells and smooth muscle cells to build a true endothelium by promoting basal lamina deposition and intercellular adhesions. The effects of rBmAsnRS observed in this study follow the same pattern.

IL-8 is a chemokine expressed by leukocytes, epithelial cells, airway smooth muscle cells and endothelial cells. Endothelial cells also store IL-8 in vesicles known as Weibel-Palade bodies [33]. The expression of IL-8 is negatively regulated by a number of mechanisms, including by the transcription factor NF-κB [34,35]. Pro-inflammatory effects of IL-8 have been well documented using leukocyte chemotaxis studies and macrophages, mast cells, neutrophils, eosinophils, lymphocytes, immature dendritic cells, and keratinocytes all express receptors that respond to IL-8. Because of the ubiquitous presence of IL-8 receptors in so many cell types, the biological activities regulated by IL-8 are equally diverse. VEGF is a potent mitogen for endothelial cells that plays a central role in angiogenesis. Both rBmAsnRS and VEGF have been shown previously to affect cellular transcription via the NF-kB pathway [17,18].

The mechanism proposed for anti-inflammatory properties of rBmAsnRS in colitis is derived from two findings: (1) the NMR structure of the 81 amino acid N terminus of rBmAsnRS showed that it folds in such a way as to mimic the way IL-8 binds to its receptor [14,15], and (2) the N terminal IL-8 like domain of rBmAsnRS is attached by a 33 amino acid flexible linker to a large catalytic region (448 amino acids) that may act as a high molecular weight “anchor” to alter cell surface receptor kinetics, receptor internalization and subsequent gene expression. Another mechanism proposed for anti-inflammatory activities mediated by IL-8 in endothelial cells is by increased expression of IL-8 receptors [36]. The quantitative effect of rBmAsnRS on expression of IL-8 receptors has not yet been investigated.

rBmAsnRS is not the first eukaryotic AARS shown to exhibit IL-8 like activity. Human tryptophanyl-tRNA synthetase (TrypRS) was shown to be cleaved by leukocyte esterase to form two separate protein, one of which has IL-8 activity and is pro-angiogenic [6,32,37]. Although both human TrypRS and filarial AsnRS are class II AARSs as defined by topology of their ATP binding sites [38], the immunologically active domains are distinct. Once human AARS were shown to be involved with angiogenesis, novel translational medical applications were developed to exploit anti-angiogenic effects in human diseases that exhibit pathology with increased vascularity [4,37]. Over the past decade, a number of clinically useful drugs have been developed that down regulate cytokine or chemokine-directed immunopathology in a variety of human diseases [39]. Therefore, as new drugs that block chemokine receptors and angiogenesis are developed for non-infectious diseases, they may also prove valuable to treat or prevent filarial pathogenesis by interference with physiocrine mediated vascular pathology.

## Supporting Information

S1 Video Widening of the blood vessel analyzed using vasodilation assay- Control. (MP4)

S2 Video Widening of the blood vessel analyzed using vasodilation assay- rBmAsnRS (10ng/ml). (MP4)

S3 Video Widening of the blood vessel analyzed using vasodilation assay- VEGF (10 ng/ml). (MP4)
