# Correction: *Brugia malayi* Asparaginyl—tRNA Synthetase Stimulates Endothelial Cell Proliferation, Vasodilation and Angiogenesis

**DOI:** 10.1371/journal.pone.0171402

**Published:** 2017-02-02

**Authors:** Jeeva Jothi D, Muthu Dhanraj, Shanmugam Solaiappan, Sanjana Sivanesan, Michael Kron, Anuradha Dhanasekaran

There are errors in the Funding section. The correct funding information is as follows: The Research was supported by two grants to AD from MHRD-CEMA F.NO-5-3/2015-TS VII and BUILDER program BT/PR12153/INF/22/200/2014. JJ acknowledges the financial support provided through the UGC-BSR fellowship sponsored by the University Grants Commission, New Delhi, India. This research was supported in part by two grants to MK from the U.S. National Institutes of Health, National Institutes of Allergy and Infectious Diseases: R21AI105472 and UO1053877.

There are errors in the Acknowledgments section. The correct Acknowledgments information is as follows: The authors greatly acknowledge Dr. Michael Kron for providing the construct. We also thank Dr. Suvro Chatterjee of Vascular Biology Lab, AU-KBC Research Centre, Anna University MIT Campus, Chennai for help with angiogenesis assays.
